# Relationship between Serum Homocysteine and Metabolic Syndrome among Patients with Schizophrenia and Bipolar Disorder: A Cross Sectional Study

**DOI:** 10.18502/ijps.v15i4.4292

**Published:** 2020-10

**Authors:** Shadi Naderyan Fe'li, Seyed Mojtaba Yassini Ardekani, Ali Dehghani

**Affiliations:** 1 Department of Biostatistics and Epidemiology, School of Public Health, Shahid Sadoughi University of Medical Sciences, Yazd, Iran.; 2 Department of Epidemiology and Biostatistics, School of Public Health, Tehran University of Medical Sciences, Tehran, Iran.; 3 Research Center of Addiction and Behavioral Sciences, Shahid Sadoughi University of Medical Sciences, Yazd, Iran.

**Keywords:** *Bipolar Disorder*, *Homocysteine*, *Metabolic Syndrome*, *Schizophrenia*

## Abstract

**Objective:** This study aimed to compare the prevalence of metabolic syndrome and hyperhomocysteinemia and to specify predictors of the metabolic syndrome among patients with schizophrenia and bipolar disorder.

**Method** **:** This cross sectional study was conducted on 100 patients with schizophrenia and 100 patients with bipolar disorder. The participants' metabolic syndrome was determined according to the criteria set by Third Report of the National Cholesterol Education Program–Adult Treatment Panel III. Hyperhomocysteinemia was considered as homocysteine levels higher than 15 µmol/L. Chi-square test, Fisher's exact test, student t test, Mann-Whitney test, and logistic regression were used for data analysis.

**Results: **The prevalence of metabolic syndrome was not significantly different (P = 0.07) between patients with schizophrenia (27%) and bipolar disorder (39%). No statistically significant difference (P = 0.17) was observed between patients with schizophrenia (82%) and bipolar disorder (74%) in the prevalence of hyperhomocysteinemia. The results of multivariable logistic regression model showed a significant association of smoking and BMI with metabolic syndrome in patients with schizophrenia (OR = 3.69, 95% CI: 1.13-12.05, and OR = 1.38, 95% CI: 1.20-1.60, respectively). In patients with bipolar disorder, BMI was a significant predictor of developing metabolic syndrome (OR = 1.29, 95% CI: 1.14-1.47). Metabolic syndrome was more prevalent in women than in men in both diagnostic groups (P < 0.05). No significant difference was observed in hyperhomocysteinemia prevalence between male and female patients with schizophrenia (P = 1.00). However, hyperhomocysteinemia was more prevalent in males than in females among patients with bipolar disorder (P = 0.001).

**Conclusion: **Findings showed a high prevalence of metabolic syndrome and hyperhomocysteinemia among patients with schizophrenia and bipolar disorder. To deal with this problem, regular monitoring and conducting early interventions are recommended to determine the metabolic risk profile and to prevent the cardiovascular diseases.

Metabolic syndrome (MetS) is a collection of metabolic disorders, including abdominal adiposity, dyslipidemia (elevated triglycerides and decreased high-density lipoprotein cholesterol (HDL-C)), hypertension, and increased fasting blood glucose (1). MetS is also a traditional risk factor and a strong predictor of developing cardiovascular disease (CVD) (1, 2).

Many recent studies reported a high prevalence of MetS among patients with schizophrenia and bipolar disorder (BD) (3-5), which can be attributed to factors like unhealthy lifestyle, limited access to health care services, and intake of antipsychotic medications (6, 7). Heald et al found that 67.6% of the patients with schizophrenia in the United Kingdom met the MetS criteria (8). Silarova et al noted that the prevalence of MetS was 28.4% among people with BD in the Netherlands (9). Moreover, in Iran, Nayerifard et al reported a high prevalence of MetS among patients with schizophrenia (28%) and BD (36%) (10). 

Homocysteine (Hcy), a nonproteinogenic amino acid, is produced during the demethylation of methionine (11). Some metabolic and nutritional deficiencies may increase the blood Hcy level (12). Medications for common medical conditions, such as lipid-lowering medications, hypoglycemic drugs, anticonvulsants, and drugs used in rheumatoid arthritis, can cause Hcy elevation (13). Beyond the traditional risk factors of CVD, such as MetS, hyperhomocysteinemia (HHcy), which is usually defined as the Hcy level of greater than 15 µmol/L (14, 15), has been proposed as a new risk factor (2, 11). In fact, increased serum levels of Hcy can be considered as an independent risk factor for developing CVD (16). In addition, Hcy and MetS may interact for the occurrence of CVD (2).

Elevated level of serum Hcy was observed in patients with schizophrenia and BD (17-19). Although reasons for the increase in blood Hcy concentration of these patients are not well known, poor nutrition, physical inactivity, smoking, and adiposity were suggested by researchers (20, 21). Moreover, HHcy can be associated with pathophysiology of schizophrenia and BD (22, 23). Mabrouk et al stated that HHcy was present in 34.4% of the patients with schizophrenia in Tunisia (24). Zhou et al reported that the prevalence of HHcy was 34.8% among Chinese patients with BD (25). Some investigators have also shown a relationship between HHcy and MetS among individuals with schizophrenia and BD. Cusa et al documented a strong association between HHcy and MetS in both patients with schizophrenia and BD (26).

The prevalence of traditional cardiovascular risk factors such as MetS have been studied largely among patients with schizophrenia and BD. However, the presence of emerging risk factors such as HHcy have been less studied in these patients. In addition, little information is available on the association of HHcy with MetS in patients with schizophrenia and BD. Due to the lack of epidemiological studies in this field, especially among Iranian patients, as well as the potential difference in genetic factors and lifestyles of various populations, this study was performed to compare the prevalence of metabolic syndrome and hyperhomocysteinemia and to specify predictors of the metabolic syndrome among patients with schizophrenia and bipolar disorder. We hypothesized whether there is a high frequency of MetS or its subcomponents and HHcy in Iranian patients. Moreover, we determined the predictors of MetS among patients with schizophrenia and BD.

## Materials and Methods

***Study Design***

This cross sectional study was performed in 2016 in Psychiatric Center and Fatemeh Al-Zahra Charity Institute in Yazd, Iran. Convenience sampling method was used and patients with diagnosed schizophrenia (n = 100) and BD (n = 100) were selected. The required sample size was calculated by the sample size formula for estimating the proportion, and considering 95% confidence level, P = 0.28, for patients with schizophrenia and, P = 0.36, for patients with BD from similar studies as well as d = 0.09. 

This study was approved by the ethics committee of Shahid Sadoughi University of Medical Sciences in Yazd, with the Ethics Code of IR.SSU.RSI.REC.1395.15. One of the participant's parents or close family members was asked to sign the informed consent form. In the case of disagreement, the patient was not entered into the study.

***Participants***

Participants were selected from the hospitalized patients whose diseases were clinically diagnosed by a psychiatrist according to the fourth edition of Diagnostic and Statistical Manual of Mental Disorders (DSM-IV-TR). Patients with schizophrenia were in the acute or chronic phase of the disease. Bipolar patients were in the manic or depressive phase of the disease or were partially treated. All participants were taking typical and atypical psychotropic medications. Inclusion criteria were having diagnosed schizophrenia or BD and Iranian nationality. Exclusion criteria included comorbidity with other psychiatric disorders, substance abuse, epilepsy, pregnancy, and mental retardation, inherited disorders of lipoprotein metabolism, chronic renal failure, and disagreement to participate in the study.

***Measurement of Study Variables***

Date of birth, available from medical records, was used for calculating the patients' age. Cigarette smoking habit (yes/no) was specified by asking the patient's nurse or family members. The administered medications, including antihypertensive, hypoglycemic, lipid-lowering drugs, and increaser or reducer Hcy agents, were investigated using the patients' medical records.

Participants were weighed by a calibrated digital scale (Omron, Japan) in light clothes and with no shoes. The patients' height was also measured in standing position and without shoes using a tape fixed on the wall. Body mass index (BMI) was calculated after dividing the weight (Kg) by height squared (m). Waist circumference (WC) was evaluated by an inelastic tape in standing position while breathing normally at the point midway between the last rib and the top of the iliac crest.

The participants' blood pressure (BP) was also measured once using a mercury sphygmomanometer (ALPK2, Japan) in sitting position from the right arm.

To measure total cholesterol (TC), triglyceride (TG), fasting blood glucose (FBG), HDL-C, and Hcy, 5 milliliter fasting venous blood sample was taken from each patient. After collecting blood specimens, they were kept at room temperature for about 30 minutes until clot formation. Later, the serum was separated by centrifugation at 2000 revolution per minute for 5 minutes. Finally, the serum samples were analyzed by an auto-analyzer (Alpha Classic analyzer, Pars Azmoon kit, Iran for TC, TG, HDL-C, FBG and Selectra E analyzer, USA, Diazyme kit, Germany for Hcy).

***Clinical Identification of the MetS and HHcy***

To define MetS, the guideline provided by the third report of the National Cholesterol Education Program Adult Treatment Panel III (NCEP ATP III) was used. The TG, BP, and FBG levels of patients who were taking triglyceride-lowering, antihypertensive, and hypoglycemic drugs were considered as abnormal (27, 28).

According to ATP III, the MetS is identified as the presence of 3 or more of the following 5 components: WC of more than 102 centimeter in men and more than 88 centimeter in women; HDL-C of less than 40 mg/dL in men and less than 50 mg/dL in women; TG ≥ 150 mg/dL and/or treatment with triglyceride lowering medications; BP ≥ 130/85 mmHg and/or treatment with antihypertensive drugs; FBG ≥ 110 mg/dL and/or treatment with glucose-lowering agents (29).

Based on the reference value of our laboratory and similar literature (17, 18, 30), HHcy was considered as Hcy of higher than 15 µmol/L.

***Statistical Analysis***

Statistical analysis of data was done by SPSS (SPSS for Windows, Chicago and SPSS Inc). The results were presented as frequency (percentage) and mean ± standard deviation to describe qualitative and quantitative variables, respectively. Chi-square and Fisher's exact tests were conducted to compare dichotomous variables. Independent sample t test or Mann-Whitney U test was also applied to compare the quantitative variables. Furthermore, P value of less than 0.05 was considered as statistically significant.

The logistic regression model was conducted to specify predictors of MetS among smoking, BMI, TC, and Hcy variables. To run the model, recommendations provided by Hosmer and Lemeshow were sued (31). In this regard, at the first step a simple logistic regression model was fitted for each risk factor, including smoking, BMI, TC, and Hcy. As a result, the risk factors that reached the modified significance level of 0.2 were retained. Later, a multiple logistic regression model was conducted on the risk factors retained from the univariate analyses and the risk factors that lost their significance were removed. Subsequently, each risk factor discarded in the univariate analyses was added to determine its significance in the multivariate model. Finally, the results of multivariable logistic model were reported. The significance level considered at this stage was 0.05. The goodness-of-fit of the final model was satisfactory.

## Results

The study population consisted of 100 patients with schizophrenia (83 males and 17 females) and 100 patients with BD (75 males and 25 females). No statistically significant difference was observed between the 2 groups in age (P = 0.63) and gender (P = 0.17) (Table 1).

No significant difference was found between the two groups regarding the prevalence of smoking, mean of BMI, WC, TC, HDL-C, FBG, and systolic and diastolic BP (P > 0.05) (Table 1). The mean of TG was significantly higher in patients with BD than those with schizophrenia (P = 0.006). Furthermore, the mean of Hcy was significantly higher in patients with schizophrenia than BD (P = 0.02).

The prevalence of MetS was 27% in patients with schizophrenia (21.7% in men and 52.9% in women) and 39% in patients with BD (33.3% in men and 56% in women). This prevalence was not significantly different between patients with schizophrenia and BD (P = 0.07). However, MetS was more prevalent in female patients than in the male patients with schizophrenia (P = 0.02) and BD (P = 0.04). Most of participants in both groups had 2 (out of 5) MetS components. With regards to schizophrenia, most female patients (35.3%) had 3 MetS components, while most males (28.9%) had 2 components of MetS (P = 0.04). Regarding BD, most females (24%) had 3 components of MetS, whereas, most males (28%) had 2 components (P = 0.25). Furthermore, low level of HDL-C was the most prevalent component of MetS in both groups. Prevalence of MetS stratified by gender showed abdominal obesity in females and low HDL-C in males were the most frequent components among participants of both groups.

The prevalence of HHcy was 82% among patients with schizophrenia (81.9% in men and 82.4% in women) and 74% among patients with BD (82.7% in men and 48% in women). The difference in prevalence of HHcy between individuals with schizophrenia and BD failed to reach the statistically significant level (P = 0.17). Among patients with schizophrenia, no significant difference was observed between males and females in HHcy prevalence (P = 1.00). However, HHcy was significantly more prevalent in males than females among patients with BD (P = 0.001). No significant difference was observed in prevalence of HHcy between users and nonusers of increaser or reducer Hcy drugs after stratification for drugs in both groups (P > 0.05). No statistically significant difference was found between patients with and without MetS in the prevalence of HHcy (P > 0.05) (Table 2).

Considering that the concentration of serum Hcy can be affected by taking several medications, a subgroup analysis was performed between participants who took Hcy drugs and those who did not with regards to the prevalence of HHcy (Table 3). The results indicated no statistically significant difference in the prevalence of HHcy based on taking increaser or reducer Hcy drugs among patients with schizophrenia and BD (P > 0.05).

The results of multivariable logistic regression analysis showed that cigarette smoking and BMI were the strongest predictors of developing MetS in patients with schizophrenia. In this vein, the odds of MetS was about 3.5 times higher in smoker patients than nonsmokers (p = 0.030). Furthermore, each one kg/m^2^ increase in BMI increased the odds of MetS by 38% (P < 0.001). In patients with BD, BMI was the best predictor of developing MetS; each one kg/m^2^ increase in BMI increased the odds of MetS by 29% (P < 0.001) (Table 4).

**Table 1:** Baseline Characteristics of Patients with Schizophrenia and Bipolar Disorder

	**Schizophrenia (n=100)** **n (%) or mean ± sd**	**Bipolar Disorder (n=100)** **n (%) or mean ± sd**	**t /M-W/χ** ^2^ ** statistic**	**P value**
Age (years)	43.11±13.80	42.23±12.18	0.48	0.63 †
Gender				
Male	83 (83)	75 (75)	1.93 (df=1)	0.17‡
Female	17 (17)	25 (25)		
Cigarette smoking habit				
Smoker	51 (51)	53 (53)	0.08 (df=1)	0.78‡
Nonsmoker	49 (49)	47 (47)		
BMI (kg/m^2^)	24.91±5.18	26.34±5.17	4279.00	0.08§
WC (cm)	93.74±15.14	95.49±14.67	4636.50	0.37§
TC (mg/dL)	167.03±43.54	171.30±39.42	4631.00	0.38§
TG (mg/dL)	150.22±89.53	174.92±93.92	3868.50	0.006§
HDL-C (mg/dL)	40.02±8.63	38.78±9.85	4399.00	0.14§
FBG (mg/dL)	87.04±12.76	92.93±32.50	4943.00	0.89§
SBP (mmHg)	114.40±16.49	113.05±17.98	4711.50	0.47§
DBP (mmHg)	77.64±11.38	76.90±10.39	4781.00	0.58§
Hcy (µmol/L)	23.87±12.12	20.67±10.06	4027.00	0.02§

BMI: body mass index, WC: waist circumference, TC: total cholesterol, TG: triglycerides, HDL-C: high-density lipoprotein cholesterol, FBG: fasting blood glucose, SBP: systolic blood pressure, DBP: diastolic blood pressure, Hcy: homocysteine.

† Independent sample t test,

‡ chi square test,

§ Mann-Whitney U test, df: degree of freedom.

**Table 2:** Difference in Hyperhomocysteinemia Prevalence between Patients with and without Presence of Metabolic Syndrome

	**Hyperhomocysteinemia**		**χ** ^2^ ** statistic**	**P value**
	**Yes (n = 156)**	**No (n = 44)**		
Schizophrenia				
MetS	24 (88.9)	3 (11.1)	1.19 (df=1)	0.38‡
Non- MetS	58 (79.5)	15 (20.5)		
All subjects	82 (82)	18 (18)		
Bipolar				
MetS	25 (64.1)	14 (35.9)	3.26 (df=1)	0.07†
Non-MetS	49 (80.3)	12 (19.7)		
All subjects	74 (74)	26 (26)		

HHcy: hyperhomocysteinemia, MetS: metabolic syndrome,

† Chi-square test,

‡ Fisher's Exact Test, df: degree of freedom.

**Table 3:** Prevalence of Hyperhomocysteinemia among Patients Treated with Increaser or Reducer Homocysteine Drugs Compared with Nonusers of These Drugs

	**Hyperhomocysteinemia**		**χ** ^2^ ** statistic**	**P value** †
	**Yes (n=156)**	**No (n=44)**		
Schizophrenia				
Increaser/reducer Hcy drugs users	38(80.9)	9 (19.1)	0.92 (df=1)	0.78
Non-users of Increaser/reducer Hcy drugs	44 (83)	9 (17)		
All subjects	82 (82)	18 (18)		
Bipolar				
Increaser/reducer Hcy drugs users	43 (78.2)	12 (21.8)	0.02 (df=1)	0.29
Nonusers of Increaser/reducer Hcy drugs	31 (68.9)	14 (31.1)		
All subjects	74 (74)	26 (26)		

HHcy: hyperhomocysteinemia, Hcy: homocysteine,

† Chi-square test, df: degree of freedom.

**Table 4:** Predictors of Metabolic Syndrome Using a Multivariable Logistic Regression Model in Patients with Schizophrenia and Bipolar Disorder

	**Adjusted OR**	**P value**	**95% CI**
Schizophrenia			
Smoking	3.69	0.030	1.13 to 12.05
BMI	1.38	<0.001	1.20 to 1.60
Bipolar			
BMI	1.29	<0.001	1.14 to 1.47

Note: each of the odds ratios were adjusted for other included variables in the model (smoking, BMI, total cholesterol, and homocysteine). MetS: metabolic syndrome, BD: Bipolar Disorder, BMI: body mass index, OR: odds ratio, CI: confidence interval.

## Discussion

The results revealed a high prevalence of MetS and HHcy in patients with schizophrenia (27%) and BD (39%). Our findings are in line with many other studies that found a remarkable prevalence of MetS in these patients (32-35). Results of a recent systematic review and meta-analysis among Indian patients with schizophrenia showed that the prevalence of MetS was 29.8% (35). The results of a meta-analysis conducted on patients with BD demonstrated that the overall MetS prevalence was 37.3% (36). Nayerifard et al reported that the prevalence of MetS was 28% and 36% among Iranian patients with schizophrenia and BD, respectively (10). The high prevalence of MetS in patients with schizophrenia and BD may be related to genetic factors, treatment with antipsychotic medications, inability to self-care, inadequate access to primary health care, and unhealthy lifestyle, such as poor eating behaviors, lack of physical activity, and smoking (8, 36-39).

Considering that all participants of this study were hospitalized and received antipsychotic agents, the high prevalence of MetS might have been due to the adverse side effects of antipsychotic agents, physical inactivity, and unhealthy diets, such as low consumption of fruits and vegetables. Furthermore, almost half of patients were smokers; smoking is effective in raising the prevalence of MetS and is a causative factor of the high frequency of low HDL-C (40). No statistically significant difference was observed between patients with schizophrenia and BD regarding the frequency of MetS, which can be due to similar characteristics among these patients, such as factors related to lifestyle, frequent admissions in hospital, and intake of antipsychotic drugs. The higher prevalence of MetS in female patients than the male was due to the fact that components of MetS were more prevalent in women (3 components) than in men (2 components). The consistency observed in the results of this research and other studies with regards to the prevalence of MetS can be due to the participants' similarity in lifestyle features, hospitalization, and use of antipsychotic drugs.

We observed that HHcy was highly prevalent in people with schizophrenia (82%) and BD (74%). The abnormal levels of Hcy were also reported by previous studies in patients with schizophrenia and BD (19, 25, 26). Kim et al found that the prevalence of HHcy was 33.8% among Korean patients with schizophrenia (41). Permoda-Osip et al reported that the prevalence of HHcy was 45% among patients with BD (17). Ezzaher et al stated that the prevalence of HHcy was 37% in Tunisian bipolar patients and it was significantly higher than healthy controls (18). The main reason of elevated Hcy level in patients with schizophrenia and BD is not exactly clear. However, some factors, such as vitamin deficiency (eg, folate, vitamin B12, or B6) caused by poor appetite, obesity, smoking, physical inactivity, and mutation in Methylen-Tetrahydrofolat Reductase gene can lead to HHcy in these patients (18, 26, 42). Also, the serum Hcy level in men can be higher than in women (18, 41). High levels of Hcy may also be related to the pathophysiology and clinical features of schizophrenia (43, 44). In addition, Hcy can be a biomarker in patients with BD (45). Moreover, some medications can increase or decrease the level of serum Hcy. For example, anticonvulsants, lithium, lipid-lowering drugs, antihypertensive, and hypoglycemic agents increase the concentration of Hcy (44, 46), but supplementation with folic acid, vitamins B12, and B6 reduce the Hcy level (11). 

The observed high prevalence of HHcy in this study may be due to the patients' inappropriate nutritional status (because of hospitalization), high frequency of smoking, high BMI, and low level of physical activity (because of hospitalization). As previously mentioned, Hcy level in men can be higher than in women (18, 41). In our study, most of the participants were male, and the high prevalence of HHcy could be due to the high frequency of male patients. In addition, elevated Hcy level is expected in these patients with regards to the role of high concentration of Hcy in the development of schizophrenia and BD. No statistically significant difference was seen in the percentage of HHcy between patients with schizophrenia and BD, which is possibly due to the similarities in the participants' lifestyle factors. Stratified analysis of the prevalence of HHcy did not show any statistically significant difference based on the use of drugs that affect the Hcy concentration. Thus, the estimated prevalence of HHcy may not be affected by the use of these drugs. In fact, HHcy prevalence may be due to factors associated with mental illness and lifestyle. 

Prevalence of HHcy was higher in our study than in others, which can be due to the differences in laboratory assay methods, genetic variations, difference between drug doses, and different dietary habits across different ethnic groups, smoking, and lack of physical activity. In addition, differences in socioeconomic status of the study populations may affect their lifestyle characteristics.

Although some previous studies on individuals with schizophrenia or BD found that HHcy was associated with the MetS or its components (19, 26, 30, 42), we found no association between MetS and Hcy. On the other hand, some other studies conducted on a general population suggested no association between HHcy and MetS. Garcin et al found no association between HHcy and MetS in a French general population (47). Budak et al confirmed the same finding in a Turkish general population (48). There are several explanations for the relationship between HHcy and MetS. HHcy is associated with components of MetS, such as hyperlipidemia, hypertension, and obesity (49). Since a random sampling method was not applied in our study, the participants may not represent the reference population entirely. In addition, we had no an opportunity to use a larger sample size. Thus, the lack of association between the study variables can be due to the applied sampling method and limited sample size. 

We observed that smoking in participants with schizophrenia and BMI in both groups played a more important role than other factors in the development of MetS. Therefore, prevention and treatment of these 2 factors should be prioritized. 

## Limitation

To the best of our knowledge, this study is the first research that investigated the prevalence of HHcy, as an emerging cardiovascular risk factor, and examined the potential association between Hcy and MetS among Iranian patients with schizophrenia and BD. However, our study had some limitations that should be considered in interpreting the results. First, the casual inferences between HHcy and MetS could not be established due to the cross sectional design and lack of temporality and a control group. Second, due to the lack of an appropriate sampling frame, we did not have the opportunity to conduct random sampling. Because of the small number of available patients, we could not use a larger sample size. Third, this study was conducted among hospitalized patients; thus, generalization of the study results to nonhospitalized patients is not recommended. Fourth, factors such as duration of illness, duration of treatment, phase of disorder, and the types and number of medications were not investigated. Finally, we did not consider some factors affecting the level of Hcy, such as inherited defects in methionine metabolism, vitamin B12, B6, and folate levels. Given that nutritional factors were not studied in this research, one cannot determine whether HHcy was caused by a diet high in methionine.

## Conclusion

Our findings suggest that inpatients with schizophrenia and BD are high-risk groups for developing MetS and HHcy. However, we observed no association between MetS and HHcy. Considering the high prevalence of MetS as a traditional cardiovascular risk factor and HHcy as an emerging risk factor of CVD among patients with schizophrenia and BD, preventive and treatment measures should be taken by psychiatrists and health care staffs. Moreover, regular checkup of the patients' physical health status, especially cardiovascular risk factors such as dyslipidemia, obesity, diabetes, hypertension, smoking, physical inactivity, unhealthy diet, and elevated level of Hcy are suggested. Prescription of appropriate antipsychotics, consideration of the patients' clinical conditions, and side effects of the drugs are also necessary.
